# Rare Hernias as Surgical Emergencies: A Case Series of Five Cases

**DOI:** 10.7759/cureus.41064

**Published:** 2023-06-28

**Authors:** Arun Singh, Shivani B Paruthy, Pramatheshwara S Aradhya, Jatin Chavda, Soni Pal

**Affiliations:** 1 General Surgery, Vardhman Mahavir Medical College and Safdarjung Hospital, New Delhi, IND

**Keywords:** amayand hernia., femoral hernia, obturator hernia, paraduodenal hernia, acute abdomen in hernia, rare hernias

## Abstract

Hernia is one of the most common clinically diagnosed cases seen in day-to-day practice. But some of them might pose a challenge in diagnosing the condition and, thus, their further management. Some types of hernias are rare and mimic the common presentation of the acute abdomen, thus requiring extra caution to keep hernias as a differential diagnosis in the acute abdomen. In this series, we report five cases of rare hernias presented to a tertiary care center in northern India over the course of one year. Two cases of paraduodenal hernias (PHs), a right and left, respectively, a male femoral hernia, an Amayand hernia, and an obturator hernia presented as acute abdomen in the emergency department, with challenges in their diagnosis, intraoperative findings, and their outcomes. Computed tomography is a useful diagnostic tool for arriving at the diagnosis pre-operatively in these situations.

## Introduction

Hernia is the protrusion of the viscus or part of the viscus through the cavity containing it. Hernia is a common case scenario in the outpatient department (OPD) and emergency room. Hernias presenting in OPD are usually uncomplicated, whereas those presenting in an emergency room can be due to obstruction, incarceration, or strangulation. They usually present with vague symptoms of pain in the abdomen and non-passage of the flatus and stools. Sometimes they have similar symptoms that recur over time. In this case series, we review a few cases of rare hernias presented to a tertiary care center in northern India. Most hernias presenting as the acute abdomen are diagnosed perioperatively, but careful observation of symptoms and relevant investigations will help in the preoperative diagnosis and better-planned management of these hernias, which will improve the prognosis in the patients. Computed tomography is a useful diagnostic tool for arriving at the diagnosis pre-operatively in these situations.

## Case presentation

Case 1

**Figure 1:**
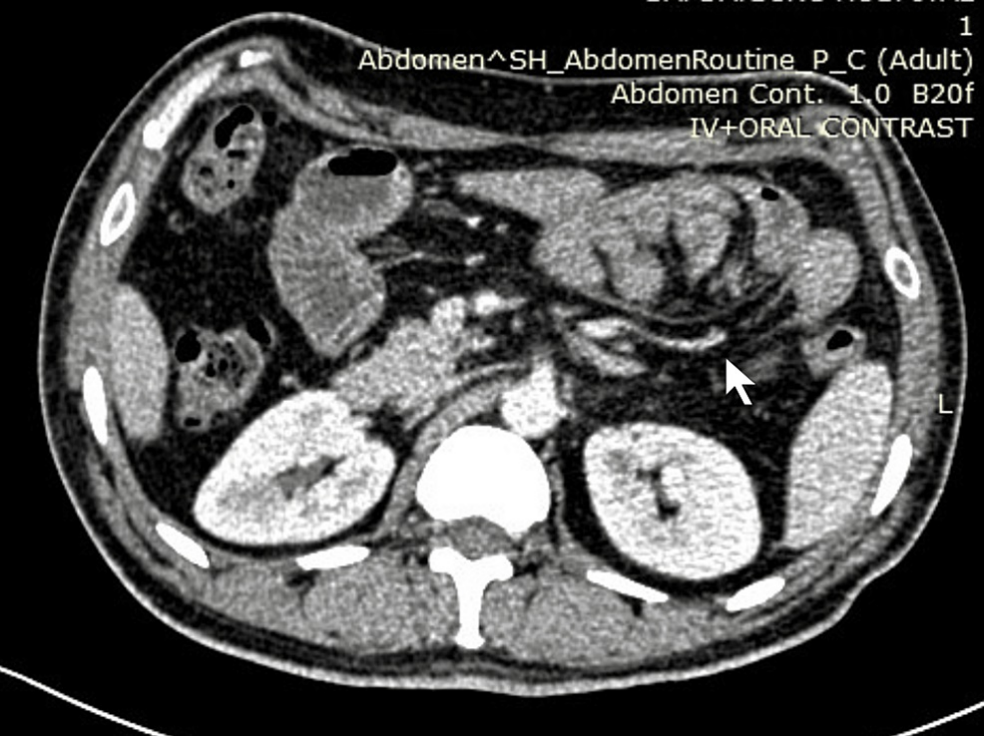
CECT abdomen: arrow showing bowel loops in paraduodenal space.

**Figure 2:**
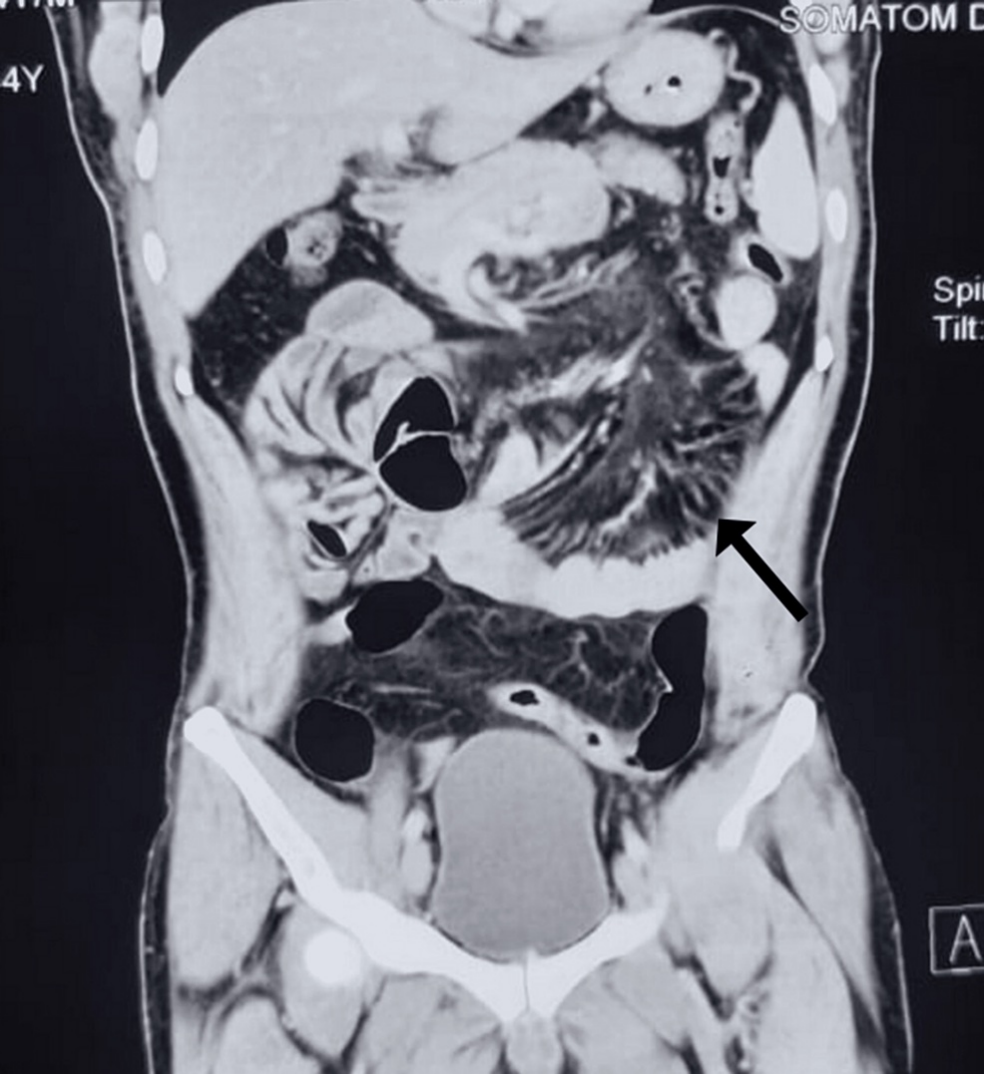
CECT abdomen showing bowel loops in paraduodenal space (arrow showing herniated ileal loops).

**Figure 3:**
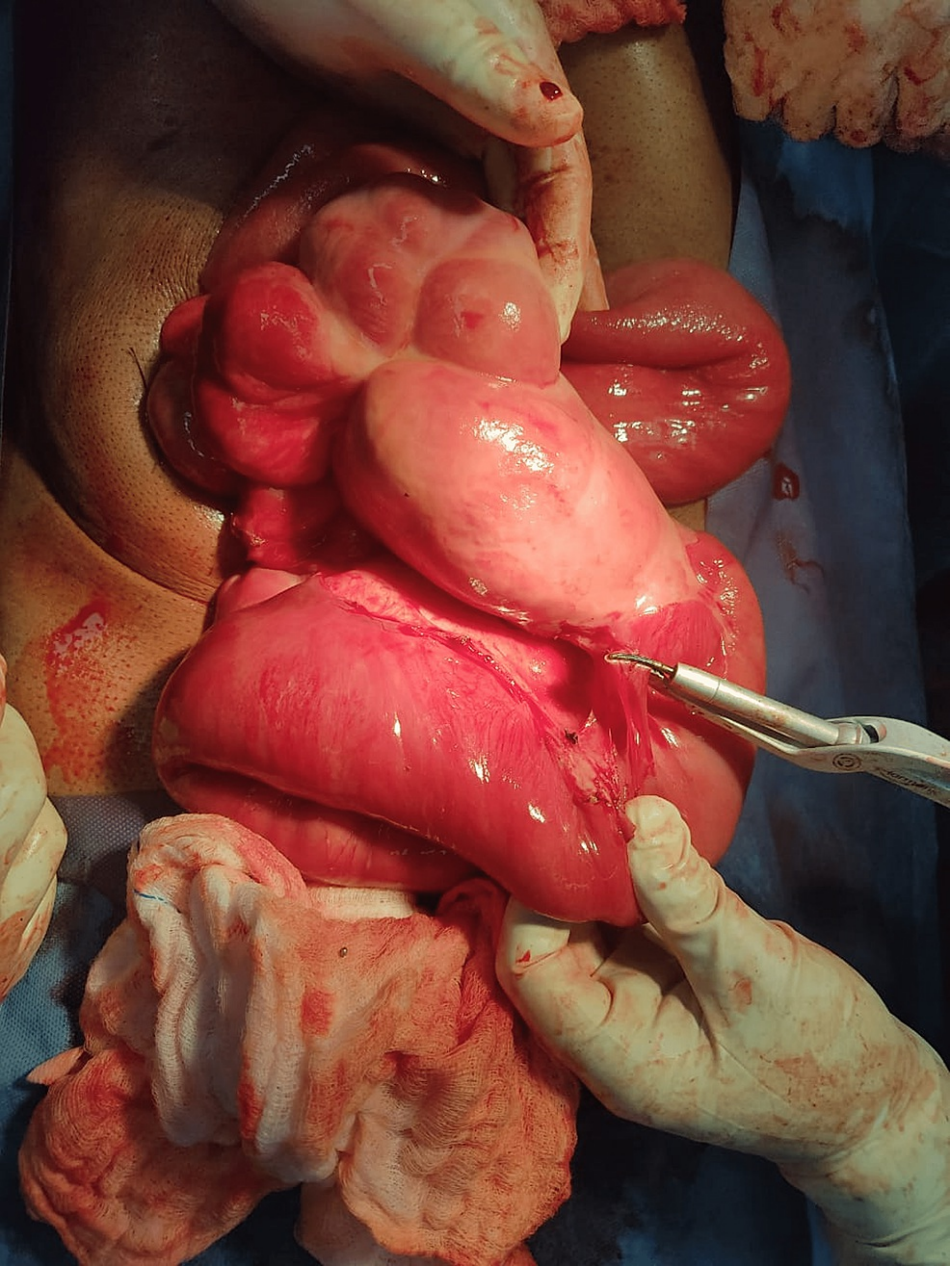
Bowel loop herniating through mesocolon.

A 44-year-old farmer from the northern part of India, a known diabetic and hypertensive, presented to emergency with a history of pain in the abdomen, which was located diffusely all over the abdomen, intermittent in nature, insidious in onset, gradually progressive colicky type, non-radiating, non-aggravating, and relieving factors associated with a history of non-passage of flatus and stools. She also complains of vomiting that is nonprojectile, bilious, and non-blood-stained. The patient had multiple similar episodes of pain, obstipation, and vomiting over eight months. Physical examination reveals the abdomen is distended, with no guarding or rigidity and absent bowel sounds. A working diagnosis of internal hernia was made based on the symptomatology and clinical examination, with no previous history of surgeries. On contrast-enhanced computed tomography (CECT) of the whole abdomen, the patient was diagnosed with a right paraduodenal hernia (PDH; Figures 1-2). Exploratory laparotomy with herniotomy and resection of the bowel with side-to-side ileo-ascending anastomosis were done. A clump of the bowel segment with a hernia is present on the right side of the abdomen. Hernial sacs densely adhere to small bowel loops. Dense adhesions are present between the gallbladder, colon, liver, and mesocolon. A defect is noted in the mesocolon, through which bowel loops pass (Figure 3). Two-and-a-half feet of the bowel (Ileum, ileocecal junction, cecum, appendix, and part of the ascending colon) are resected. Ileo-ascending colon anastomosis was done with a 75-mm stapler. The post-operative period was uneventful, and the patient was discharged on post-operative day (POD) 6.

Case 2

**Figure 4:**
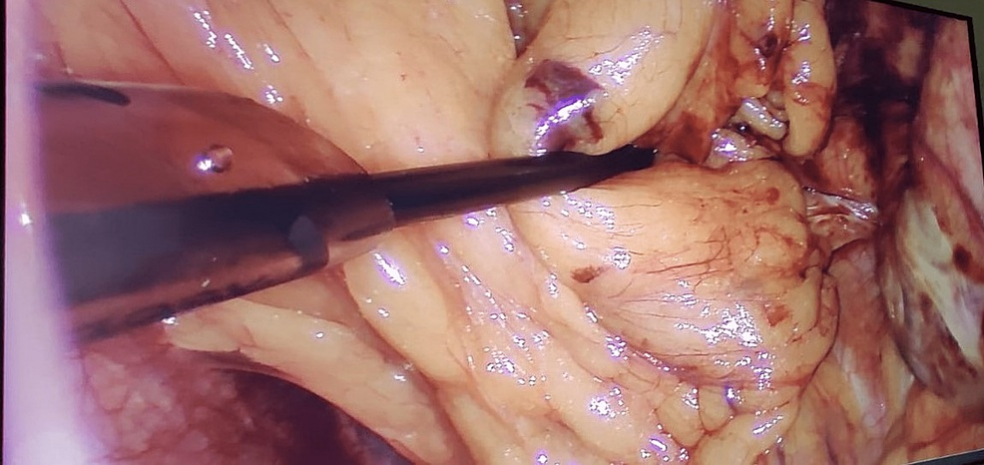
Laparoscopic view showing dense adhesions.

**Figure 5:**
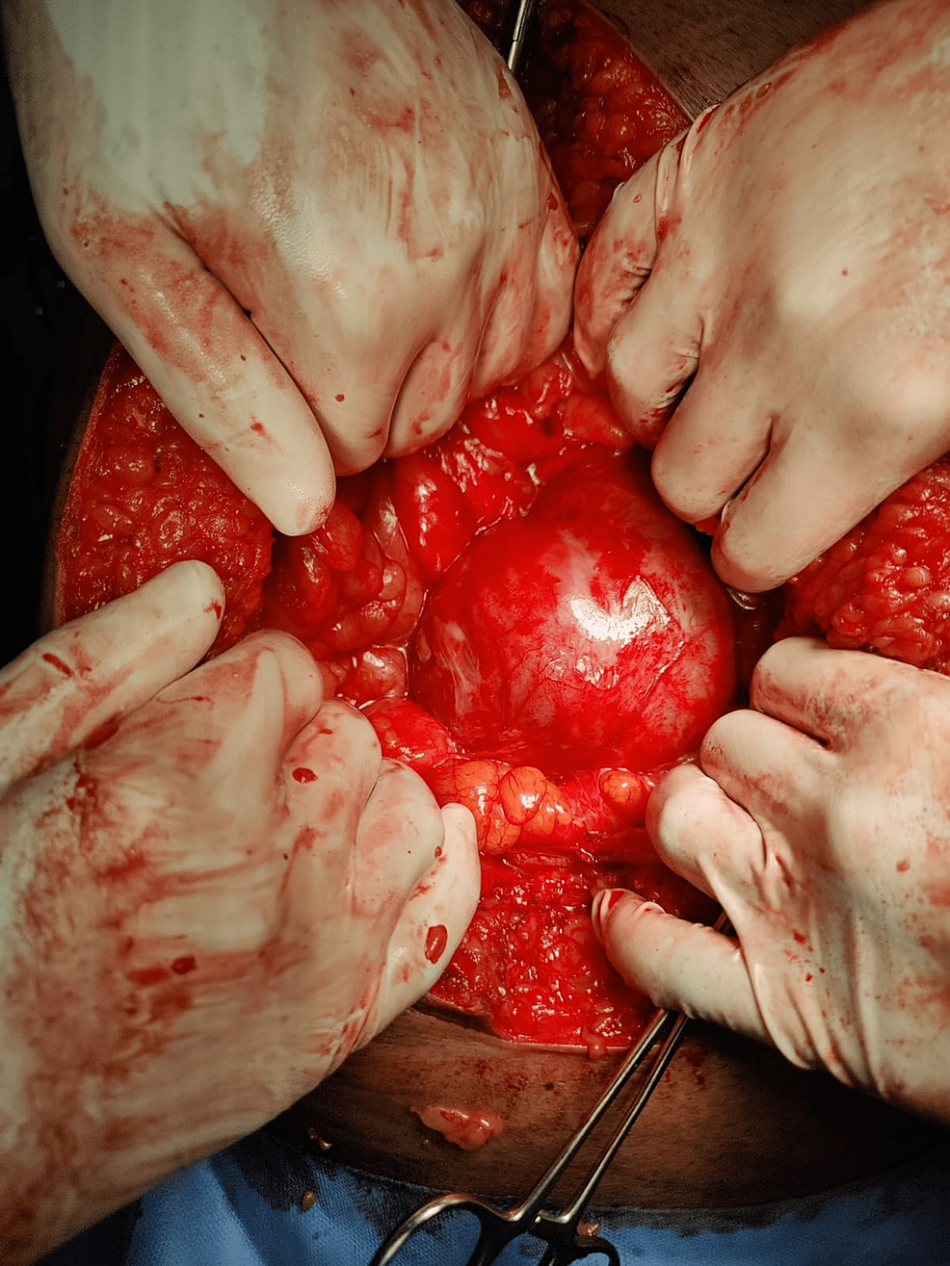
Left paraduodenal hernia in Landzert’s fossa - showing clumped bowel loops within hernial sac.

A 38-year-old known hypertensive and diabetic male shopkeeper presented to emergency with pain in the abdomen, which was of the colicky type, non-radiating, aggravated by eating food and lying down, relieving on taking medication, and intermittent on and off episodes. Also, complaints of nausea and vomiting that were non-projectile, bilious in nature, and non-blood-stained contained ingested food particles. On physical examination, the abdomen is distended, tense, and mildly tender, with no guarding/rigidity noted and absent bowel sounds. The patient had multiple episodes of similar events, which subsided after Ryle's tube insertion. The patient also has multiple episodes of presentation to the ER with complaints of not passing flatus and stools, which were managed conservatively, and the symptoms were relieved after a day or two. Laparoscopic hernia repair was tried (Figure 4), but the procedure was abandoned, and open exploratory laparotomy with anatomical repair was done because of dense adhesions present between the bowel loops and the parietus. Intraoperatively, the left paraduodenal hernial sac was noted with small bowel loops of the jejunum and ileum as content. A defect was noted in the transverse mesocolon with dense adhesions of the bowel to the sac in the transverse mesocolon. The whole small bowel was clumped and formed a cocoon in the hernial sac of approximately 20 cm × 15 cm in size (Figure 5). The post-operative period was uneventful. On POD-2, the patient started passing flatulence and stools and was discharged on POD-5.

Case 3

**Figure 6:**
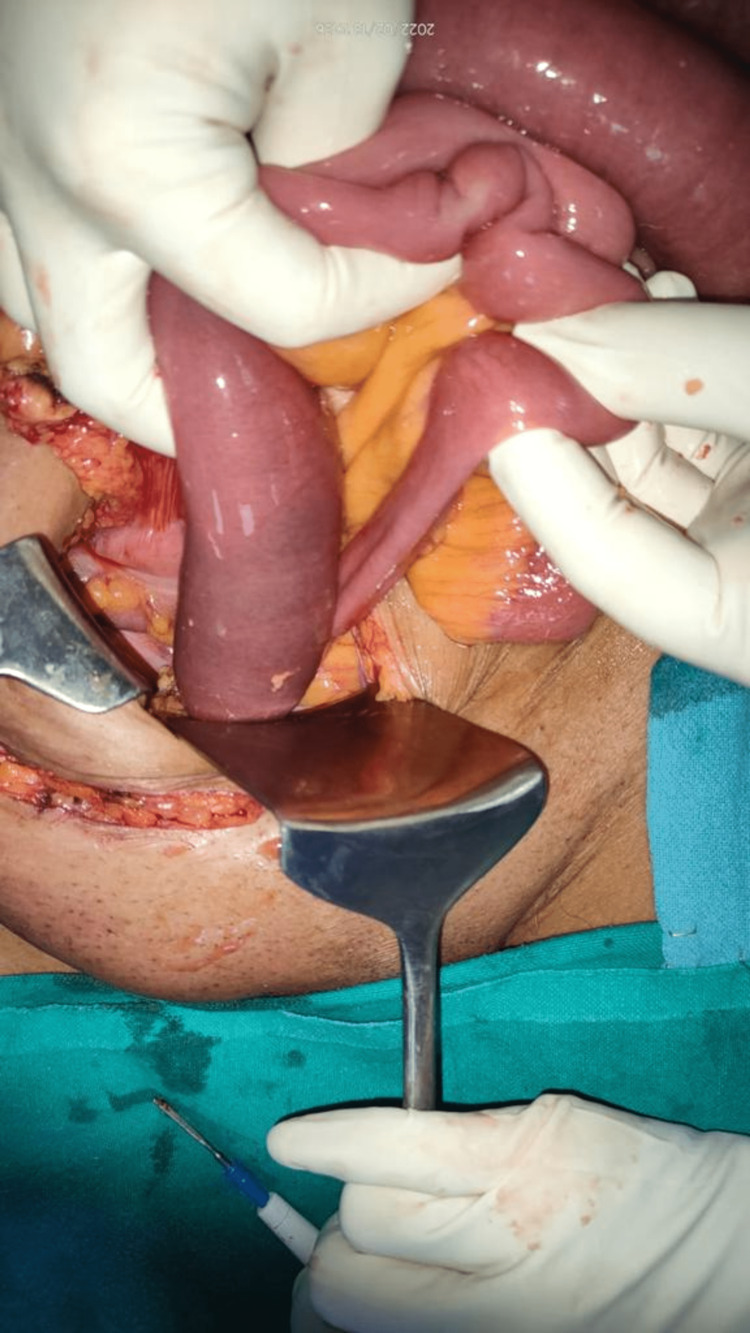
Femoral hernia-bowel loop in femoral canal.

A 75-year-old male farmer from the northern part of India presented to the emergency room with symptoms of swelling in the right upper thigh close to the inguinal ligament for four months. Associated with pain described as colicky, intermittent, non-radiating, aggravated on eating food, and relieved on lying down. His previous medical history reveals hypertension, and he is on regular anti-hypertensive medications. Physical examination of the mass reveals a reducible swelling in the upper thigh below and lateral to the pubic tubercle with a positive cough impulse. A working diagnosis of femoral hernia was made based on the symptomatology and clinical examination. The patient underwent the Mcevedys procedure, where an incision was placed directly over the femoral canal, extending to the inguinal ligament directly upwards, and the sac was dissected from below. An intraoperatively strangulated bowel loop was seen through the femoral canal (Figure 6), and resection and anastomosis were done. The post-operative period was uneventful. On POD-3, the patient started passing flatulence and stools and was discharged on POD-5.

Case 4

**Figure 7:**
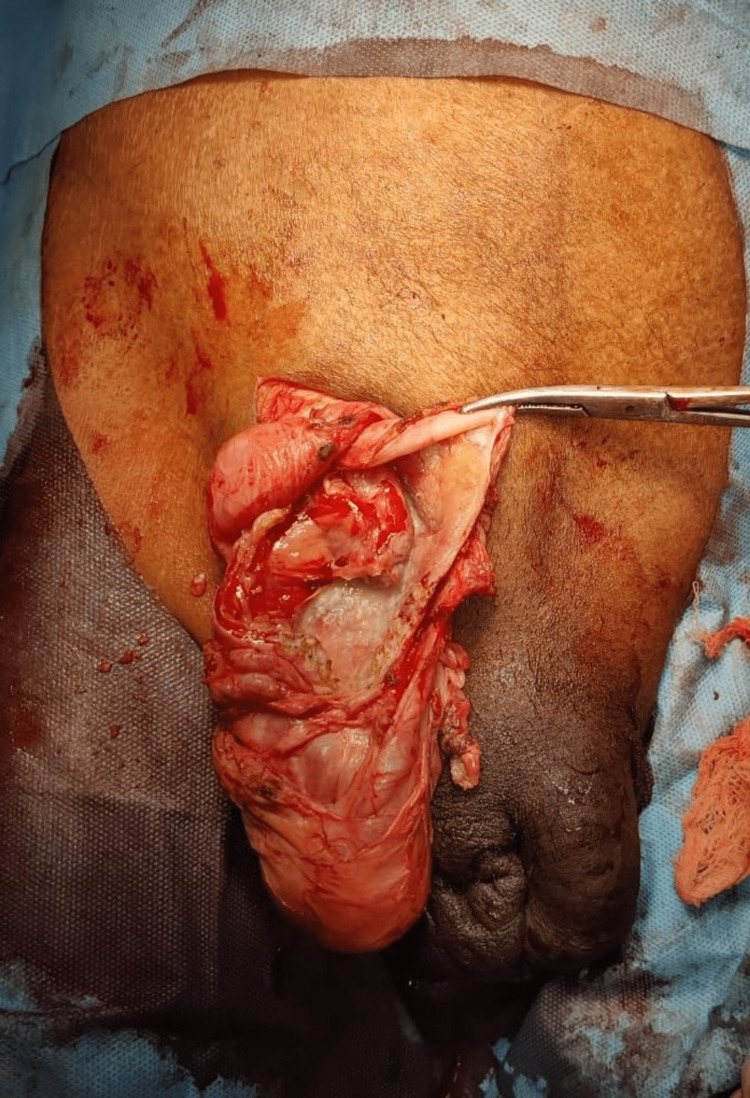
Right amayands hernia - right inguinal canal showing appendix.

A 47-year-old farmer from the northern part of India presented to the emergency room with painful swelling in the right groin region. The patient has had swelling in the right groin region for two years but started developing pain in the last two days. Associated nausea, vomiting, and altered bowel movements are present. A history of smoking and lifting heavy weights is present. On physical examination, partially reducible swelling is noted in the right inguinal region, which is tender with reddish discoloration of the overlying skin. On exploration intraoperatively, a dilated and inflamed appendix was noted as the content of the hernial sac (Figure 7). The base of the appendix was normal. The appendectomy was done, the peritoneum was closed, and the modified Bassini's repair was done by suturing the conjoint tendon with the inguinal ligament with polypropylene interrupted sutures. Mesh placement was not done. The postoperative period was uneventful, and the patient was discharged on POD-4.

Case 5

**Figure 8:**
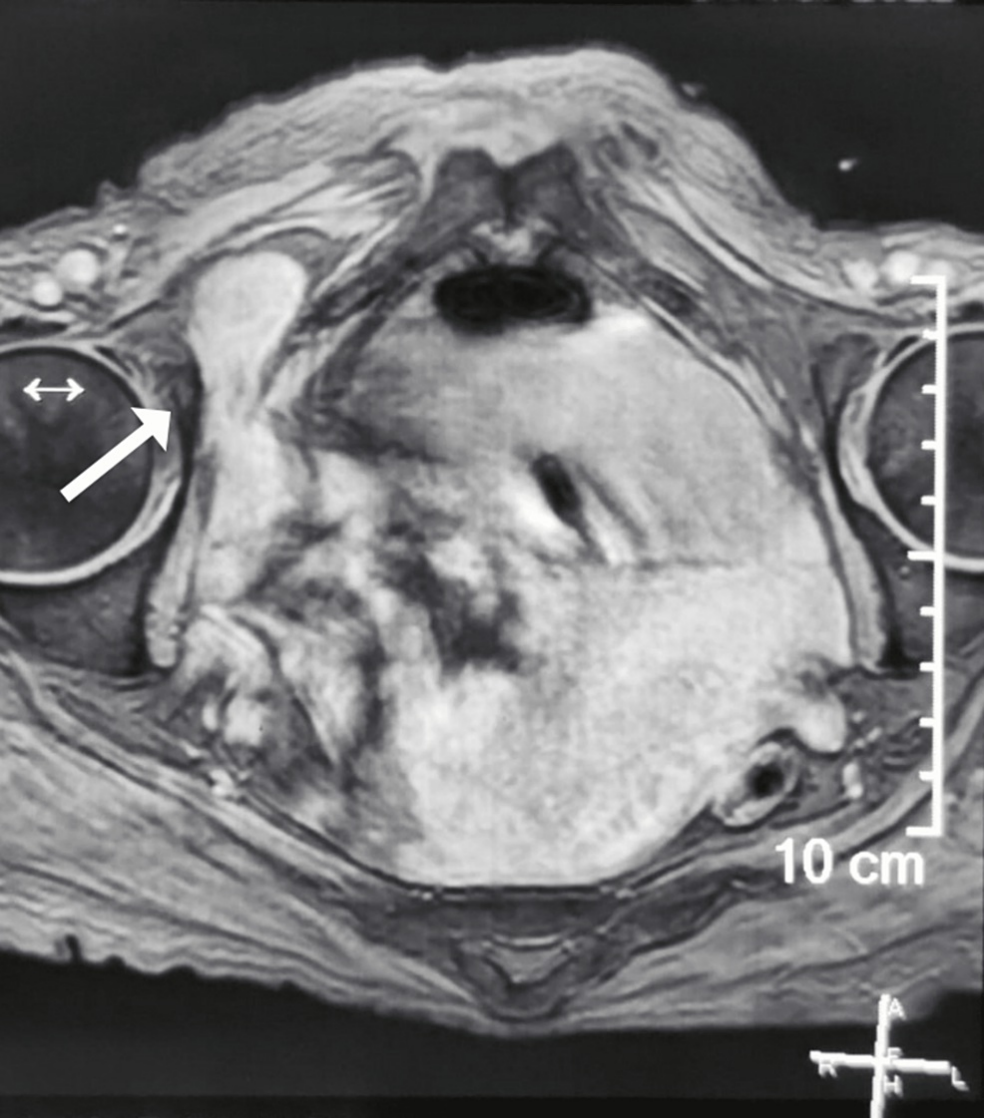
Magnetic resonance imaging: arrow showing bowel loop in right obturator canal.

**Figure 9:**
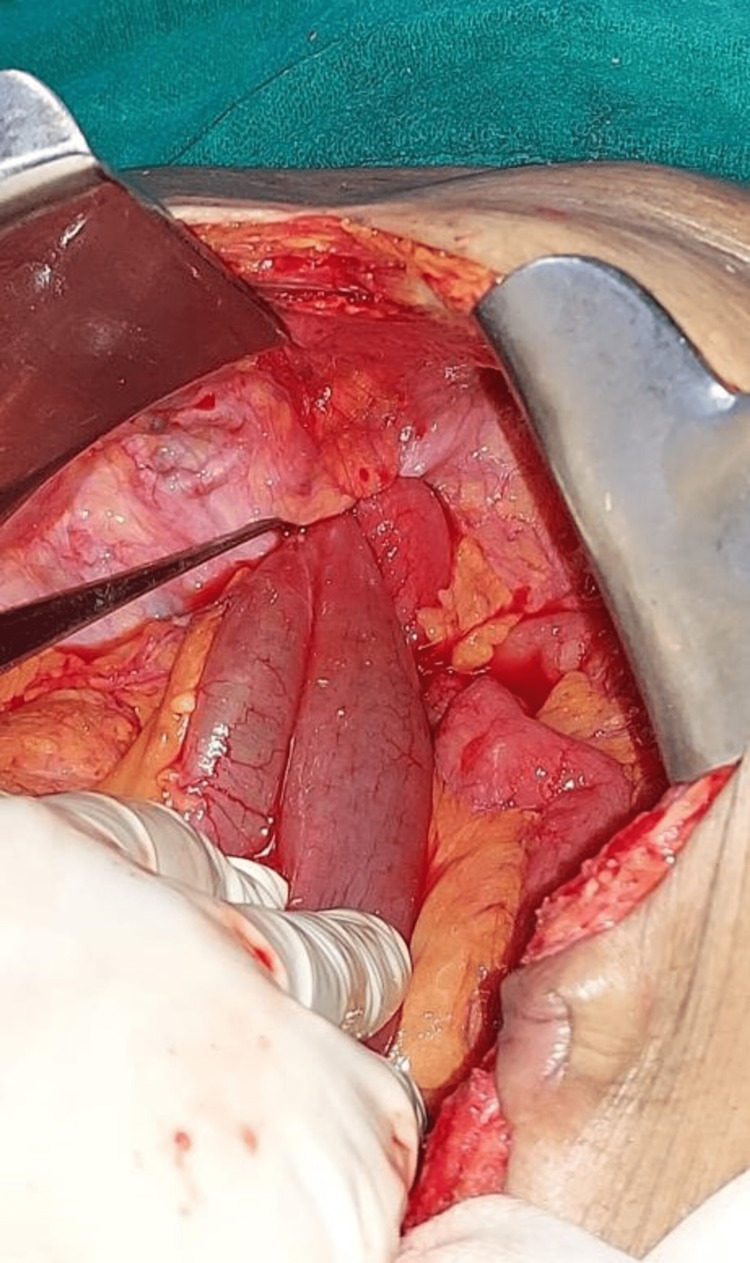
Right obturator hernia showing bowel loop in obturator canal.

An 81-year-old female housewife from north India presented to the emergency room with not being able to pass flatus and stools, nausea, vomiting, and associated pain in the abdomen, which was located in the right lower quadrant, was insidious in onset, gradually progressive, radiating to the right buttock, aggravated the movement of the right leg, and relieved on taking medications. On palpation, tenderness was present, but no mass was palpable in the right iliac fossa. Blood parameters and an ultrasound examination were inconclusive. On MRI, a small bowel loop was seen herniating into the obturator foramen on the right side (Figure 8), thus diagnosing it as a right obturator hernia. Later, the patient was taken into emergency OT, and the abdomen was explored through a lower midline incision. Ileal loops herniating into the obturator foramina were noted (Figure 9), and after the reduction of ileal loops and confirming their viability defect was repaired, the peritoneum closed. The postoperative period was uneventful and was discharged on POD-5.

Listed below is the consolidated list of all cases (Table 1).

**Table 1:** Consolidated list of all cases.

Patient	Age (years)	Sex	Presenting complaints	Type of hernia	Procedure done
Case 1	44	M	Multiple episodes of pain abdomen and obstipation	Right paraduodenal hernia	Anatomical repair
Case 2	38	M	Multiple episodes of pain abdomen and obstipation	Left paraduodenal hernia	Anatomical repair
Case 3	75	M	Swelling in the upper thigh and pain abdomen	Right femoral hernia	Mc Evedy’s procedure
Case 4	47	M	Groin pain swelling and pain in abdomen	Right amayand hernia	Appendectomy with modified Bassinis repair
Case 5	81	F	Pain abdomen, nausea, and vomiting	Right obturator hernia	Exploratory laparotomy with the anatomical repair

## Discussion

Hernia is defined as an abnormal protrusion of the viscus or part of the viscus through the cavity containing it. Hernias can be one of the differential diagnoses for patients presenting with acute abdominal pain. Obstruction, incarceration, or strangulation are the causes of hernias presenting as acute abdominal pain. Inguinal hernia and umbilical hernia are common causes of acute abdomen; careful monitoring is needed to prevent complications.

Internal hernias are a well-known but rare cause of intestinal obstruction, accounting for 0.2% to 0.9% of cases [1]. PDH is the most common type of internal hernia. Among PDH, left-sided PDH accounts for 75% of PDH, and the rest is right-sided PDH [2]. Abnormalities in this physiological rotation, in the second stage, lead the pre-arterial segment to rotate posteriorly and to the left of superior mesenteric artery (SMA), leading to invagination of the bowel into the descending mesocolon near the fourth part of the duodenum, forming the fossa of Landzert, leading to the left paraduodenal hernia. Thus, the herniated small bowel lies in a sac with anterior margins formed by the descending mesocolon, ascending branch of the left colic artery, and inferior mesenteric vein [3,4]. The right paraduodenal hernia occurs when bowel loops migrate through the defect in the mesentery of the first part of the jejunum, called Waldeyers fossa. Right PDH is found on the right side of the transverse mesocolon and extends inferolaterally behind the ascending mesocolon [5,6]. Clinical features of PDH can range from nausea, vomiting, epigastric discomfort, abdominal pain, and distension to chronic postprandial pain and recurrent episodes of acute intestinal obstruction. Unresolved obstruction might lead to dire complications like ischemia and perforation. The diagnosis of PDH is often missed, as internal hernias are usually overlooked. Barium follow-through can be a useful investigation in the diagnosis of PDH; however, a CT scan is the investigation of choice in modern-day practise [2]. The difficulty in diagnosing PDH is further multiplied by the transient nature of herniation, which requires repeated CTs for diagnosis. Surgically, the aim is to reduce the hernia, restore the anatomy, and repair the defect. In left-sided PDH, care must be taken to avoid damage to the Left colic artery and Inferior mesenteric vessels, which are found in the anterior part of the hernial opening, and in right-sided PDH, care must be taken to avoid damage to the superior mesenteric vessels.

A femoral hernia occurs through the femoral canal. The femoral canal is an inverted cone-shaped, 1-2 cm-long fascial space present in the medial part of the thigh. Anteriorly made of the medial part of the inguinal ligament, posteriorly made of the pectineal ligament overlying the pectineus muscle and its fascia covering the superior pubic ramus, laterally made of the femoral vein within the intermediate compartment of the femoral sheath, and medially made of the lacunar ligament [7]. Although the most common hernia in females is still an inguinal hernia, femoral hernias are more common in females than males [8]. Females are at higher risk than males, as females have a broader pelvis, giving them a chance for a larger femoral canal and providing space medially. Elderly age and positive family history are also very important independent risk factors for femoral hernia development [9]. Patients with a femoral hernia usually present with pain in the lower abdomen or upper thigh, along with swelling in the upper thigh region. These patients remain apparently asymptomatic until they present with features of the acute abdomen [10], and it was also noted that patients who had undergone an emergency procedure for an incarcerated femoral hernia first presented with symptoms to healthcare within the week before hospital admission [11]. In older females, femoral hernias usually present as incarcerated hernias [12]. Therefore, conservative management is not advised, and upfront surgery is the choice for elderly females [8]. A femoral hernia can be managed by a laparoscopic approach using transabdominal preperitoneal repair (TAPP) or transabdominal extraperitoneal repair (TEP) [8] or an open preperitoneal mesh placement technique.

Protrusion of the vermiform appendix in the inguinal hernia sac with or without appendicitis is known as Amayand's hernia [13]. Claudius Amayand, a French surgeon, was the first person to perform an appendectomy successfully on an 11-year-old boy who presented with an inflamed and perforated appendix in the hernial sac in 1735 [14]. Amayand's hernia usually occurs on the right side due to the normal anatomical position of the vermiform appendix. Rarely, it can occur on the left side in cases of situs inversus, intestinal malrotation, or mobile caecum [15]. Different surgical modalities can be used for Amayand's hernia. Appendicectomy is recommended for an inflamed appendix without using mesh. The use of mesh for hernia repair in contaminated wounds is advocated by some surgeons but strongly opposed by others due to the high possibility of post-operative wound infection [16]. The presence of prosthetic material will increase the inflammatory response; hence, the use of mesh is disregarded in infective cases.

Among abdominal hernias, the incidence of obturator hernias is less than 1% [17]. Preoperative diagnosis is rarely done. This type of rare hernia usually presents in a surgical emergency with features mimicking acute intestinal obstruction. It is usually seen in elderly, emaciated females [18], owing to the loss of fat in the peri-obturator space area, which causes an increased chance of herniation along with a larger obturator canal in females than males. It is usually seen on the right side since the left-sided foramina is guarded by the sigmoid colon. The diagnosis of obturator hernia is usually made on contrast-enhanced CT scans, which show bowel loops protruding through the obturator canal between the obturator internus and obturator externus muscles. Obturator hernias can be managed by minimally invasive surgery or as an open procedure. In an elective setting, the patient can be managed by TAPP or TEP, but in an emergency setting, a conventional lower midline incision has an advantage as it helps in better dissection and exposure and complete control of the disease process [19].

## Conclusions

Though hernias are very common cases seen in routine practise, we should be aware of some rare hernias presenting as acute abdomens requiring immediate action. When required, a thorough history and clinical examination adjunct with CECT abdomen should suffice. Internal hernias, along with the rare presentation of other hernias, should be a differential diagnosis in the acute abdomen. Early, timely intervention can curtail disease progression and increase survival.
